# Plant Viruses in Plant Molecular Pharming: Toward the Use of Enveloped Viruses

**DOI:** 10.3389/fpls.2019.00803

**Published:** 2019-06-19

**Authors:** Ahmad Ibrahim, Valerie Odon, Richard Kormelink

**Affiliations:** Laboratory of Virology, Department of Plant Sciences, Wageningen University & Research, Wageningen, Netherlands

**Keywords:** plant molecular pharming, non-enveloped viruses, plant rhabdoviruses, recombinant vaccines, virus like particles, biotechnology

## Abstract

Plant molecular pharming has emerged as a reliable platform for recombinant protein expression providing a safe and low-cost alternative to bacterial and mammalian cells-based systems. Simultaneously, plant viruses have evolved from pathogens to molecular tools for recombinant protein expression, chimaeric viral vaccine production, and lately, as nanoagents for drug delivery. This review summarizes the genesis of viral vectors and agroinfection, the development of non-enveloped viruses for various biotechnological applications, and the on-going research on enveloped plant viruses.

## Introduction, Plant Viruses From Pathogens to Biological Toolkit

Virology began in 1892 with D. Ivanovsky’s paper describing the retention of virulence in leaf sap extracted from Crimean tobacco with mosaic leaf disease (Lustig and Levine, 1992). Even though the extracted sap had been passed through bacteria-retaining filters, the filtrate retrained the ability to replicate within living plants (Beijerinck, 1898). Later, Vinson (1927) succeeded in precipitating the pathogen of tobacco mosaic disease, and experiments in 1936 by Bawden and Pirie revealed that this pathogen contained RNA and protein components (Bawden et al., 1936; Bawden and Pirie, 1937). Further experiments in 1956 demonstrated that genetic information was stored in RNA molecules (Fraenkel-Conrat et al., 1957) and later the concept of self-assembly of RNA and coat protein (CP) into particles was established (Butler and Klug, 1971). This pathogen is known as tobacco mosaic virus (TMV) and is one of the prominent viruses in plant molecular pharming.

**FIGURE 1:**
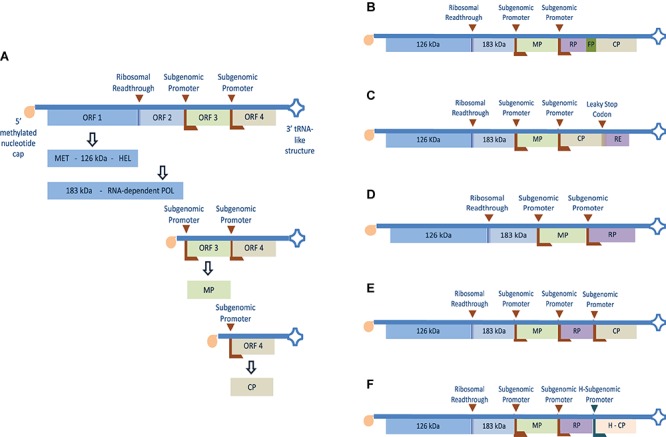
Genomic organization and expression strategy of TMV and different strategies adapted to express recombinant protein or heterologous epitope using the full virus genome. **(A)** The positive single-stranded RNA genome has four separate ORF(s) with a 5′ terminus methylated nucleotide cap (m7G5’pppG) and 3′-terminus tRNA-like structure. The first two 5′ proximal ORF(s) encode 126 and 183 kDa readthrough proteins containing methyltransferase (MET), helicase (HEL), and polymerase (POL) domains which are involved in replication and transcription of the genome. ORF 3 and 4 are translated from separate subgenomic promoters and encode the 30 kDa movement protein (MP) and 17 kDa coat protein (CP) respectively. **(B)** Recombinant protein (RP) fused to the CP N-terminus using fusion peptide (FP) (Roder et al., 2017). **(C)** Recombinant epitope (RE) fused to the CP C-terminus using leaky UAG stop codon (Sugiyama et al., 1995). **(D)** Coding region of RP cloned in place of the virus CP gene (Takamatsu et al., 1987). **(E)** Coding region of RP downstream a subgenomic RNA promoter and placed between the MP and the CP genes (Dawson et al., 1989). **(F)** Coding region of RP cloned downstream TMV subgenomic RNA promoter and placed between TMV MP gene and a heterogeneous CP (H-CP) gene. The latter was cloned into the TMV vector together with its heterogeneous subgenomic promoter from odontoglossum ringspot virus (ORSV) (Donson et al., 1991).

Tobacco mosaic virus belongs to the *Tobamovirus* genus with a positive sense, single-stranded genomic RNA (gRNA) with a 7-methylguanosine-5-triphosphate cap at the 5′ terminus (Dunigan and Zaitlin, 1990) and a 3′ untranslated region (UTR) harboring a transfer RNA (tRNA)-like structure (Takamatsu et al., 1990) as shown in Figure 1A. It encodes a total of four proteins two of which are involved in RNA replication plus a movement protein (MP) and a CP (Goelet et al., 1982). Inside the plant cell, the gRNA acts as a messenger RNA (mRNA) template for expressing a 126 kDa protein containing methyltransferase and helicase domains plus a 183 kDa (readthrough) protein containing a polymerase domain (Osman and Buck, 1996; Lewandowski and Dawson, 2000). The 126 kDa and the 183 kDa replication proteins bind to the terminal tRNA-like structure initiating transcription of complementary (negative-sense) template (Lewandowski and Dawson, 2000; Osman and Buck, 2003). This negative-sense RNA acts as a template for the synthesis of full-length positive strands and subgenomic RNAs containing MP and CP open reading frames (ORFs) (Ishikawa et al., 1991). The MP is an RNA binding protein involved in cell-to-cell spreading of the virus (Citovsky et al., 1990; Chen et al., 2000) while the CP enhances the formation of replication complexes (Asurmendi et al., 2004), long-distance movement (Saito et al., 1990; Hilf and Dawson, 1993), and viral particle assembly (Bloomer et al., 1978; Butler, 1999).

During the 1980s, the field of plant molecular pharming was born (Franken et al., 1997) and various pharmaceuticals such as human hormones (Barta et al., 1986), antibodies (Hiatt et al., 1989), and vaccines (Thanavala et al., 1995), were produced using transgenic plants. Currently, several proteins manufactured in plants are commercialized such as bovine trypsin TrypZean expressed in maize and commercialized by Sigma-Aldrich (#T3568, Sigma-Aldrich Corporation, United States), the 2006 USDA approved Newcastle disease virus for poultry produced in tobacco cell-suspension by Dow AgroSciences (Vermij and Waltz, 2006), and the 2012 FDA approved taliglucerase alfa (Elelyso^®^) for the management of type 1 Gaucher’s disease produced in carrot cells by Protalix Biotherapeutics Inc. (Maxmen, 2012).

However, the generation and selection of stably transformed plants for heterologous protein expression is quite elaborate and time-consuming which lead scientists to research exploiting viruses for this purpose. Plant viruses such as TMV (Takamatsu et al., 1987) and cowpea mosaic virus (CPMV) (Gopinath et al., 2000) were first adapted as full-virus vector then as a deconstructed-virus vector for recombinant protein expression. Later on, as we understood more of viral structure and capsid assembly, non-enveloped plant viruses were also manipulated for the generation of chimeric viral particles, nanoagents for carriage of various compounds and nanostructures building blocks. Furthermore, recently enveloped plant viruses, such as rhabdoviruses, have been recovered from agroinfiltrated cDNA, adding a viral particle with an amendable lipid envelope to the arsenal of available systems.

## Plant Viruses in Recombinant Expression Technology, First-Generation Vectors

Adopting plant viruses as vectors for transient expression offered many advantages over the transgenic system such as ability for application in various plant species (Hamamoto et al., 1993), reduced gene-to-product time (Hendy et al., 1999), and increased yields (Maclean et al., 2007). At first, the host cells were infected with a full copy of plant virus (either DNA or *in vitro* transcribed RNA) into which the heterogenous sequence or gene of interest (GOI) was cloned. The first published virus-based vector was a gene-replacement model in which the GOI replaced the CP of brome mosaic virus (BMV) (French et al., 1986). However, lacking the CP, the recombinant virus was unable to spread, and infection was limited to inoculated cells. Further attempts followed using TMV-based vectors in which the GOI was inserted upstream the inherent CP gene and controlled by an additional subgenomic RNA promoter (the inherent CP subgenomic promoter was duplicated) (Dawson et al., 1988). Still this design was unstable and homologous recombination resulted in the loss of inserted sequence and reversion to the wild-type virus (Dawson et al., 1989). A third approach was to create a hybrid virus-vector containing different subgenomic promoter sequences from two tobamoviruses [TMV and odontoglossum ringspot virus (ORSV)]. This hybrid design resulted in a more stable vector that succeeded in the systemic expression of the recombinant protein (Donson et al., 1991). Other designs included fusing the recombinant protein to the TMV CP either at the C-terminal (Roder et al., 2017) or at the N-terminal downstream a leaky stop codon (Sugiyama et al., 1995). Figures 1B–F, show the above described modification approaches applied to TMV. Table 1 lists a number of plant-made pharmaceutical proteins expressed using full-viral vectors.

**TABLE 1:** Plant-made proteins expressed using full viral vectors.

**Full virus strategy**			
**Recombinant protein**	**Viral vector**	**Delivery method**	**References**
Amyloid β protein (Aβ) fragments	Cucumber mosaic virus (CMV)	Mechanical inoculation of *in vitro* transcribed RNA	Vitti et al.,2010
Cholera toxin b subunit	Tobacco mosaic virus (TMV)	Mechanical inoculation of *in vitro* transcribed RNA	Moore et al.,2016
Dihydrofolate reductase (DHFR)	Cauliflower mosaic virus (CaMV)	Mechanical inoculation of naked DNA	Brisson et al.,1984
Human anti-non- Hodgkin’s lymphoma single-chain Fv (scFv) immunoglobulins	Hybrid tobacco mosaic virus (TMV) and odontoglossum ringspot virus (ORSV)	Mechanical inoculation of *in vitro* transcribed RNA	McCormick et al.,2003
Rice α-amylase	Hybrid tobacco mosaic virus (TMV) and tomato mosaic virus (ToMV)	Mechanical inoculation of *in vitro* transcribed RNA	Kumagai et al.,2000
Capsid protein VP1 of foot-and-mouth disease virus (FMDV)	Bamboo mosaic virus (BaMV)	Mechanical inoculation of naked DNA	Yang et al.,2007

## Plant Viruses in Recombinant Expression Technology, Second-Generation Vectors

**FIGURE 2:**
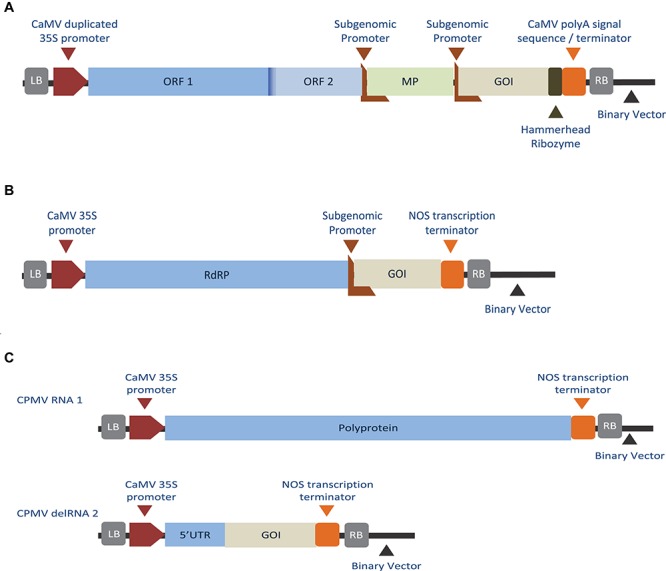
Diagrams showing different replicon-based expression systems. **(A)** Deconstructed monopartite RNA virus, TMV-based system, where the viral CP was substituted with the GOI under the control of a subgenomic promoter (Lindbo, 2007). **(B)** Deconstructed monopartite RNA virus, PVX-based system, where the viral CP and MP were substituted with the GOI under the control of a subgenomic promoter (Komarova et al., 2006). **(C)** Deconstructed bipartite RNA plant virus, CPMV-based system, where the RNA-1 component supplies the viral RdRp, virus genome-linked protein, helicase, and protease, while the modified RNA-2 contains the GOI, replacing those of the viral MP and CP (Cañizares et al., 2006). **(A)** Described replicon design is capable of replication, cell-to-cell movement, transcription, and replication, while designs **(B,C)** are capable of all, but deficient in cell-to-cell movement. LB, left border; RB, right border of the T-DNA region.

Although viral vectors based on a full-genome demonstrated success in producing recombinant proteins, constructs with large inserts showed instability and low systemic spread (Shivprasad et al., 1999; Avesani et al., 2007). Together with biosafety concerns (Scholthof et al., 1996), these limitations lead to the development of second-generation vectors in which the virus genome was deconstructed into a replicon containing the essential viral genomic components for replication and gene expression while plant-infection was initiated exogenously. In this system, viral MP and/or CP genes were replaced by the recombinant GOI and the vector was introduced into plants as part of *Agrobacterium* delivered T-DNA (Gleba et al., 2004), biolistic bombardment (Komarova et al., 2006) or as chromosome-inserted replicon (Cañizares et al., 2006). Lacking the CP, the deconstructed-virus based vectors lacked the ability to encapsidate into viral particles, however, they retained the ability to replicate, transcribe, and translate as shown in Figure 2.

Among the monopartite RNA viruses, potato virus X (PVX) (Komarova et al., 2006) and TMV (Lindbo, 2007) were first to be adopted as a deconstructed-virus vectors. Deconstructed-TMV based vectors were further commercially pursued by Icon Genetics as magnifection technology (trademarked as magnICON^®^) (Gleba et al., 2005). The modifications included using a hybrid RdRP [from turnip vein-clearing virus (TVCV)] and *Arabidopsis* actin 2 (ACT2) as a promoter together with removal of cryptic thymine-rich intron sites plus selective introduction of introns (Marillonnet et al., 2004, 2005). Magnifection of hybrid TMV-based vectors yielded 4 g/kg of fresh weight tissue (FWT) of recombinant protein and 4.8 g/kg FWT of full immunoglobulin G (IgG) in less than 2 weeks (Bendandi et al., 2010).

Similarly, a deconstructed virus strategy was developed for the bipartite RNA1/RNA2 CPMV. Early research was based on maintaining an unmodified RNA-1 while introducing the recombinant gene to the RNA-2 construct. Recombinant expression was achieved using this methodology with both full-length and defective versions of RNA-2 coding plasmids (Liu et al., 2005). Surprisingly, vector systems based on deleted regions of RNA-2 (delRNA-2) achieved higher expression yields than those obtained with full-length RNA-2 vectors (Sainsbury et al., 2008). Further experiments revealed that replication of delRNA-2 based molecules was not essential and high yields of recombinant protein expression was still achieved in the absence of RNA-1 (Sainsbury et al., 2008). The modified delRNA-2-based mRNA was “hyper-translated” providing approximately 0.3 g/kg FWT of fully assembled monoclonal antibody within 6 days after agroinfection (Sainsbury and Lomonossoff, 2008). Table 2 lists a number of plant-made pharmaceutical proteins expressed using deconstructed-viral vectors.

**TABLE 2:** Plant-made proteins expressed using deconstructed viral vectors.

**Deconstructed virus strategy**			

**Recombinant protein**	**Viral vector**	**Delivery method**	**References**
Assembled full-size monoclonal antibody	Cowpea mosaic virus (CPMV)	Agroinfiltration of plasmids encoding viral vectors	Sainsbury and Lomonossoff,2008
Assembled full-size monoclonal antibody	Combination of non-competing viral vectors tobacco mosaic virus (TMV) and potato virus X (PVX)	Agroinfiltration of pro-vector modules for *in planta* assembly	Giritch et al.,2006
Green fluorescent protein (GFP)	Cucumber mosaic virus (CMV)	Agroinfiltration of plasmids encoding viral vectors	Fujiki et al.,2008
Hepatitis B core Norwalk virus capsid protein (NVCP)	Bean yellow dwarf virus (BeYDV)	Agroinfiltration of BeYDV-derived vector with viral replication-protein supplying vector	Huang et al.,2009
Human growth hormone	Hybrid crucifer-infecting tobacco mosaic virus (cr-TMV) and turnip vein-clearing virus (TVCV)	Agroinfiltration of pro-vector modules for *in planta* assembly	Gils et al.,2005

The CPMV RNA2-based expression system allowed an elegant extension in which the genome-integrated cDNA of RNA-2 was amplified using agroinfiltrated RNA-1 constructs or by crossing with RNA-1 transgenic plants (Cañizares et al., 2006). This method of an inducible-replicon system, or dormant viral cassette, was also applied with a tomato mosaic virus (ToMV) system in suspension-cultured plant cells (Dohi et al., 2006). Other applications included development of dormant viral cassettes which can be activated at a custom-chosen stage using chemical inducers, such as ethanol (Zhang and Mason, 2006), or via a Cre-*LoxP* recombination system (Tremblay et al., 2007).

## Plant Viruses as Biological Particles, the Case of Non-Enveloped Viruses

**FIGURE 3:**
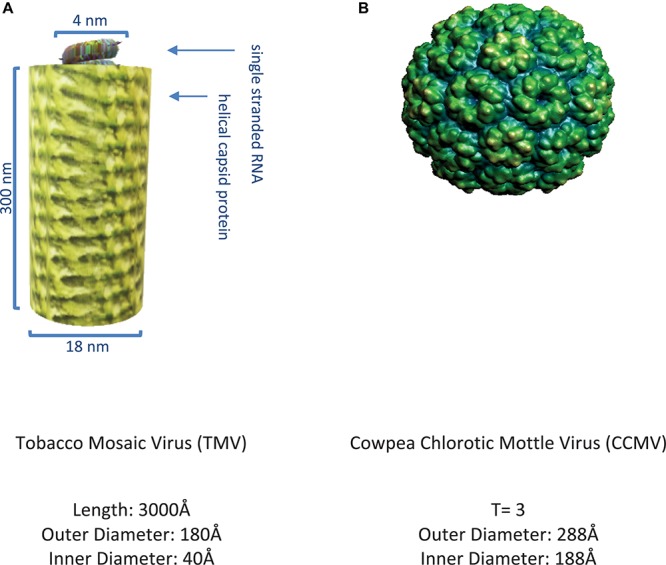
Representative plant viruses. **(A)** Image of tobacco mosaic virus (Namba and Stubbs, 1986) rendered on MS PowerPoint. **(B)** Image of cowpea chlorotic mottle virus (Speir et al., 1995) as obtained from VIPER (Carrillo-Tripp et al., 2009).

The plant viruses first exploited in biotechnology were non-enveloped and consist of CP subunits that have the ability to self-assemble into filamentous structures such as TMV (Alonso et al., 2013) or hollow symmetric icosahedral structures such as CCMV (Zandi et al., 2004) as shown in Figures 3A,B.

**FIGURE 4:**
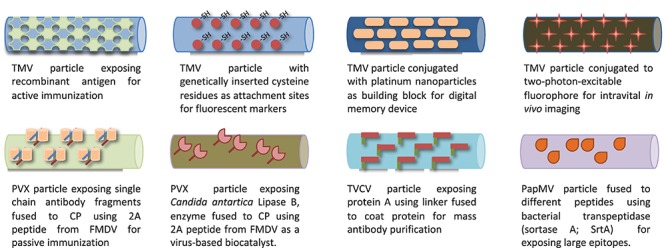
Various filamentous plant viruses engineered for different biological applications.

With better understanding of particle architecture, biophysical properties, and ability to manipulate their genomic material, these viruses also became exploited as virus-like particles (VLP), self-assembled structures devoid of any genomic material (Huang et al., 2009) and further developed as virus-based nanoparticles (VNP) (Steinmetz et al., 2009). For instance, the detailed molecular structure knowledge of filamentous viruses such as TMV (Namba et al., 1989), PVX (Parker et al., 2002), TVCV (Lartey et al., 1994), and PapMV (Yang et al., 2012) permitted their use for various applications such as vaccines (Smolenska et al., 1998; Petukhova et al., 2013; Therien et al., 2017), fluorescent markers (Yi et al., 2005), biocatalysts (Carette et al., 2007), nanoparticles for biologics purification (Werner et al., 2006), nanoparticles for *in vivo* imaging (Niehl et al., 2015), and assembly units for memory devices (Tseng et al., 2006), as shown in Figure 4.

Non-enveloped plant viruses have been engineered in three different ways: modification of the outer capsid (whether through genetic manipulation, chemical modification of capsid’s amino acids moieties, or through a combination of both), incorporation of heterologous cargo in the inner cavity, or particle integration into multivalent structures. Table 3 provides examples of some current VNP that are being developed as agents used in gene delivery (Azizgolshani et al., 2013), chemotherapy (Sánchez-Sánchez et al., 2014), immunotherapy (Venuti et al., 2015), vaccines (Phelps et al., 2007), and plant virus-assisted sensors (reviewed in Eiben et al., 2018).

**TABLE 3:** List of some of the non-enveloped plant viruses that have been produced for VLP or VNP applications.

***Utilizing the exterior surface of viral particles (VLPs as scaffolds for peptide/epitope presentation)***			

**Virus**	**Viral structure**	**Type of modification**	**References**
Alfalfa mosaic virus (AMV)	Icosahedral	Plant-produced chimaeric VLP as vaccine for rabies virus and human immunodeficiency virus.	Yusibov et al.,1997
Cowpea chlorotic mottle virus	Icosahedral	Genetic manipulation and chemical modification for multivalent presentation of ligands	Gillitzer et al.,2002
Cowpea mosaic virus	Icosahedral	Plant-produced chimaeric virus vaccine for human rhinovirus 14 and human immunodeficiency virus	Porta et al.,1994
Papaya mosaic virus	Rod-Shaped	*E. coli* produced VLP as chimaeric viral vaccine for influenza virus.	Rioux et al.,2012
Potato virus X	Rod-Shaped	Plant-produced chimaeric virus vaccine for hepatitis C virus.	Uhde-Holzem et al.,2010
Red clover necrotic mosaic virus	Icosahedral	Plant-produced VLP developed for targeted drug delivery.	Lockney et al.,2010
Tobacco mosaic virus	Rod-Shaped	Plant-produced chimaeric virus vaccine for influenza virus.	Petukhova et al.,2013
Turnip yellow mosaic virus	Icosahedral	Plant-produced VLP developed as a biological probe.	Barnhill et al.,2007

***Utilizing the inner cavity of the virus***			

**Virus**	**Viral structure**	**Note**	**References**

Brome mosaic virus	Icosahedral	Plant-produced VLP developed for encapsulation of organic chromophore.	Jung et al.,2011
Cowpea chlorotic mottle virus	Icosahedral	*E. coli* produced VLP developed for encapsulation of polymers.	Douglas and Young,1998
Cowpea mosaic virus	Icosahedral	Plant-produced VLP developed for encapsulation of metals.	Aljabali et al.,2010
Hibiscus chlorotic ringspot virus	Icosahedral	Plant-produced VLP developed for drug delivery.	Ren et al.,2007
Tobacco mosaic virus	Rod-Shaped	Plant-produced VLP developed for drug delivery.	Czapar et al.,2016

Production of plant virus based VLPs as vaccines received the attention since the early 1990 (Usha et al., 1993). Plant RNA viruses such as TMV (Koo et al., 1999), CPMV (Mclain et al., 1995), CMV (Nuzzaci et al., 2007), plum pox potyvirus (PPV) (Fernández-Fernández et al., 1998), potato virus X (PVX) (Lico et al., 2009), and tomato bushy stunt virus (TBSV) (Joelson et al., 1997) have been adapted for the production of vaccines and tested in various animal models. Studies conducted on the biodistribution and clearance of TMV (Bruckman et al., 2014), PVX and tomato bushy stunt virus (TBSV) (Blandino et al., 2015) in animal models showed no VLP induced pathological damage. Plant viruses are not pathogenic to mammals but are recognized by the pathogen associated molecular pattern (PAMP) receptors of the innate immune system (Acosta-Ramírez et al., 2008). They were shown to elicit a humoral response when administered by parenteral (Brennan et al., 1999b) or mucosal routes (Brennan et al., 1999a) and a cell mediated response (Yusibov et al., 2005; Kemnade et al., 2014). For instance, PapMV-CP-M2e VLP can induced murine anti-M2e antibodies that recognized influenza-infected cells and provided 100% protection (Denis et al., 2008) without any PapMV VLP induced cellular toxicity (Rioux et al., 2014).

The production of plant virus based VLP(s) were not limited to plants, and a variety of heterologous systems such as insect cells (Lamb et al., 1996), yeast (Brumfield et al., 2004) or *Escherichia coli* (Denis et al., 2008) have been employed to produce recombinant potato leafroll virus (PLRV), CCMV, and PapMV CP that assemble into viral particles indistinguishable from their plant produced counterparts. Nevertheless, plant-produced VLPs remain the most economical choice, as plant production is scalable and cost-effective with production costs lower than those in *E. coli* or eukaryotic-based systems (Kusnadi et al., 1997; Tusé et al., 2014), and has substantial safety advantage compared to mammalian-cells produced alternatives (Ma et al., 2003).

## Toward Enveloped Virus Like Particles

In the last decade, VLPs have been extensively developed as recombinant vaccines in different systems whether in plants or mammalian (Soulie et al., 1991), insects (Le Tallec et al., 2009), yeast (Agnandji et al., 2011), and *E. coli* (Fiers et al., 2009). They have proven to be immunogenic and few are currently available on the market such as Engerix^®^ [for hepatitis B virus (HBV)] (Keating and Noble, 2003), and Cervarix^®^ [for human papillomavirus (HPV)] (Szarewski, 2010), produced by GlaxoSmithKline, or their respective equivalents Recombivax HB^®^ (Venters et al., 2004), and Gardasil^®^ (Tomljenovic and Shaw, 2012) produced by Merck.

Nevertheless, producing VLPs whether through genetic fusion or chemical conjugation remains challenging, especially for complex multimeric antigens (Jagu et al., 2013; Benen et al., 2014), full length glycoproteins that adopt different conformations (Kwong et al., 2002; Cullen et al., 2017), large epitopes (Werner et al., 2006), or have termini that are involved in protein folding (Pejawar-Gaddy et al., 2010) and might interrupt the capsid synchronized assembly (Zlotnick, 1994; Roldão et al., 2012), while post-expression chemical conjugation has the drawback of further increasing downstream processing costs (Buonaguro and Butler-Ransohoff, 2010; Wilken and Nikolov, 2012; Sabalza et al., 2014; Buyel et al., 2015). Furthermore, chimeric VLPs expressing recombinant peptides were found to induce reduced neutralizing antibodies titre when compared to the full domain vaccines (Gedvilaite et al., 2015). Even among plant-virus based VLP(s), for instance, alfalfa mosaic virus-based *Plasmodium falciparum* Pfs25 VLP, although it was shown to induce *P. falciparum* blocking antibodies in mice (Jones et al., 2013), in Phase I clinical trial it was shown to provide a low protection level (Chichester et al., 2018).

In line with those difficulties and limitations, and considering that some of these are not encountered with enveloped VLPs (eVLPs), there has been a growing trend toward the use of eVLPs as production platform (Gheysen et al., 1989). In this system, structural viral proteins are expressed and incorporated into host membranes released as particles. The envelope provides the flexibility for integration of complete envelope-proteins and glycoproteins resembling the native virus (Yamshchikov et al., 1995; Baumert et al., 1998; Latham and Galarza, 2001). Along this idea attempts have been made to produce various eVLPs for vaccine purposes, e.g., against influenza virus (production of eVLPs in insect cells expressing influenza virus structural proteins) (Khurana et al., 2011), HIV (based on virosome, *in vitro* associated/spiked with an HIV1 gp41-derived peptide) (Leroux-Roels et al., 2013) and breast cancer (based on *in vitro* association of antigenic peptides to synthesized phospholipid membranes) (Wiedermann et al., 2010).

Influenza eVLPs have been produced from the co-expression of the two major antigenic envelope proteins hemagglutinin (HA) and neuraminidase (NA) plus matrix 1 (M1) (Pushko et al., 2005) or from the co-expression of HA and M1 to enable budding (Galarza et al., 2005). Most of these attempts have been performed in animal cell systems, but recently plants have also been used as platform for producing eVLPs against avian H5N1 Influenza (Landry et al., 2010). HA-based influenza VLPs were found to be budding from the plasma membrane of plant cells expressing HA only, without the need of further viral proteins (D’Aoust et al., 2008), due to the absence of glycoprotein sialylation in plants (Séveno et al., 2004). Moreover, and importantly, these plant-produced HA based eVLP vaccines were found to elicit durable and cross-reactive T cell responses and are currently undergoing clinical trials by Medicago (Landry et al., 2014). These findings support and strengthen the development and exploitation of enveloped plant virus-based vector systems, amenable for genetic manipulation, in plant molecular pharming. Few plant viruses exist that have a naturally occurring envelope. They classify within the orders *Bunyavirales* and *Mononegavirales*, of which the family *Rhabdoviridae*, and have a negative strand RNA genome (King et al., 2018). Considering that monopartite rhabdoviruses are easier to handle and a reverse genetics system has recently been established for a plant infecting rhabdovirus, they present the most attractive platform for development of pseudotyped viral-vaccines upon which their inherent envelope will provide the needed flexibility to incorporate large and complex antigens.

## Plant Viruses as Biological Particles, the Case of Inherently Enveloped Viruses – Rhabdoviruses as an Example

**FIGURE 5:**
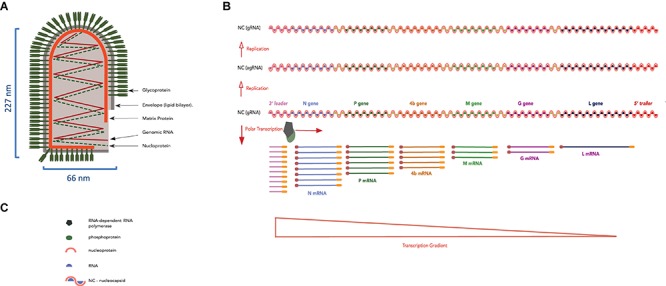
LNYV bullet-shape structure and its genomic RNA organization, replication and transcription strategy. **(A)** Diagram representing an LNYV particle showing the transmembrane glycoprotein, lipid bilayer, matrix protein, and genomic RNA together with the nucleoprotein forming the ribonucleoprotein coil. **(B)** LNYV gRNA (negative-sense) showing the 3′ *leader*, the N, P, 4b, M, G, and L genes, the 5′ *trailer* (sequential genes separated by a gene junction) and the transcription gradient. Genomic RNA is first replicated into agRNA (positive-sense), which is used as template for the production of (accumulating amounts of) progeny gRNA (negative-sense). **(C)** Legend of panel **(B)**.

The *Rhabdoviridae* family contains an ecologically diverse group of viruses infecting hosts from a wide plethora of aquatic and terrestrial vertebrates and plants (Kuzmin et al., 2009); rhabdoviruses from different kingdoms are listed Table 4. Plant rhabdoviruses, like all other viruses of the *Rhabdoviridae* family are enveloped, negative-sense RNA viruses (Jackson et al., 2005). They have a bacilliform shape defined by two major structural components: an outer envelope made of the host lipid bilayer embedded with surface projections of the virus glycoprotein and a tightly coiled internal nucleocapsid composed of genomic RNA (gRNA) together nucleoprotein, phosphoprotein, and the polymerase protein (Wolanski et al., 1967) (Figure 5, traditionally, plant rhabdoviruses were divided into *Cytorhabdovirus* or *Nucleorhabdovirus* genera, depending on the virus propagation sites within the cell while recently two new genera *Dichorhavirus* and *Varicosavirus*, with bipartite genomes, were added (Dietzgen et al., 2017; Whitfield et al., 2018).

**TABLE 4:** List of some rhabdoviruses including plant *Cytorhabdoviruses* and *Nucleorhabdoviruses* together with their host, host class, and vectors.

***Plant Rhabdoviruses***				

**Cytorhabdovirus**				

**Virus**	**Plant host and class**		**Vector**	**References**
Barley yellow striate mosaic virus	*Hordeum vulgare* and*Triticum durum*	Monocotyledonous	*Laodelphax striatellus*	Conti,1969
Lettuce necrotic yellows virus	*Lactuca sativa*	Dicotyledonous	*Hyperomyzus lactucae*	Stubbs and Grogan,1963

**Nucleorhabdovirus**				

Cereal chlorotic mottle virus	*Zea mays*	Monocotyledonous	*Nesoclutha pallida*	Greber,1979
Sonchus yellow net virus	*Sonchus oleraceus* and*Bidens pilosa*	Dicotyledonous	*Aphis coreopsidis*	Christie et al.,1974

***Animal Rhabdoviruses***				

**Vesiculovirus**				

**Virus**	**Animal host**		**Vector**	**References**

Vesicular stomatitis Indiana virus	Livestock		*Lutzomyia shannoni*	Tesh et al.,1972
Vesicular stomatitis New Jersey virus	Livestock		*Simulium vittatum*	Comer et al.,1990

**Lyssavirus**				

Rabies virus	Broad host range		*Vamplire bats*	Mollentze et al.,2014
Duvenhage virus	Broad host range		*Insectivorous bats*	Tignor et al.,1977

***Fish Rhabdoviruses***				

**Virus**	**Fish host**		**Vector**	**References**

Snakehead rhabdovirus	*Ophicephalus striatus*		*–*	Ahne et al.,1988
Rhabdovirus of penaeid shrimp	*Penaeus stylirostris*		*–*	Lu et al.,1991

Many of the plant rhabdoviruses are transmitted by arthropods in a persistent propagative way in which the virus enters and replicates within the insect before transmission (Sylvester and Richardson, 1992; Creamer et al., 1997; Redinbaugh et al., 2002) while dichorhaviruses are transmitted by mites (Dietzgen et al., 2018) and varicosaviruses by soil-borne fungi (Latham et al., 2004). Their spread is correlated with that of the vector (Sylvester and Richardson, 1992), and no seed transmission has been reported. They infect both monocotyledonous (Conti and Appiano, 1973) and dicotyledonous (Salazar et al., 2000) plants, harming agricultural production (Chen et al., 1979; Fang et al., 1996). The typical symptoms of viral infection are necrosis, mosaic mottling of leaf tissue, and vein clearing (Jackson et al., 2009).

Plant rhabdovirus gRNA encodes the rhabdoviruses canonical genes: Nucleoprotein (N), Phosphoprotein (P), Matrix (M), Glycoprotein (G), and Large Polymerase (L) (Jackson et al., 2018), in addition to accessory genes (Walker et al., 2011) such as those coding for a cell-to-cell MP such as the 4b gene in lettuce necrotic yellow virus (LNYV) (Dietzgen et al., 2007), gene 3 of rice yellow stunt rhabdovirus (RYSV) (Huang et al., 2005). Furthermore, the gRNA is flanked by UTRs termed 3′ *leader* and 5′ *trailer* (Fu, 2005) (Figure 5B). The *leader* and *trailer* sequences are partially complementary and contain *cis*-acting signals involved in transcription and replication (Zuidema et al., 1986; Choi et al., 1994; Wang et al., 1999) while short intergenic sequences regulate mRNA synthesis and sequential transcription of the canonical genes (Wetzel et al., 1994; Bandyopadhyay et al., 2010).

### Rhabdoviruses Transcription

Theoretical models that explain rhabdovirus transcription and replication were built upon experiments on mammalian rhabdoviruses, and one of the most well studied is Vesicular Stomatitis virus (VSV) (Emerson, 1987; Wertz et al., 1987). The ribonucleoprotein (RNP) unit, consisting of viral RNA associated with the N, L, and P proteins, acts as the template for viral transcription and replication (Yang et al., 1998, 1999; Kawai et al., 1999). Virion-associated L protein transcribes the NC into mRNA of distinct proteins (Moyer and Banerjee, 1975; Toriyama and Peters, 1980). The transcription direction and gradient follow the order of genes in the virus genome and are controlled by the untranslated 3′ – *leader* sequence (Emerson, 1982) and the non-translated inter-genomic regions respectively as shown in Figure 5B (Barr et al., 1997; Finke et al., 2000). Transcription of the viral genes results in the generation of monocistronic 5′-capped and polyadenylated mRNAs.

### Rhabdoviruses Replication

Later during the infection, the N-P complex inhibits transcription and switches the function of the L protein from that of transcription to replication (La Ferla and Peluso, 1989; Gupta and Banerjee, 1997). First, the gRNA is replicated into an antigenomic sense RNA (agRNA) template, which is full positive-sense RNA without a cap and poly (A) tail and is encapsidated by the N protein (Arnheiter et al., 1985; Zhang et al., 2008). Later, agRNA functions as a template for progeny negative-sense gRNA (Albertini et al., 2008; Ivanov et al., 2011). With the progeny gRNA assembled as RNP, the M protein converts the extended helical RNP into a condensed form (Kaptur et al., 1991), resulting in the bullet-shape morphology of rhabdoviruses (Mebatsion et al., 1999). Finally, the G protein tail is incorporated into the assembled M-RNP complex, aiding the budding of progeny virus particles (Whitt et al., 1989; Schnell et al., 1998).

### Recovery of Rhabdoviruses From cDNA

Initial attempts to generate rhabdoviruses from cDNA were based on mammalian rhabdoviruses. Research on VSV revealed that genomic RNA must be encapsidated with the viral N protein to be a functional template for RNA-dependent RNA polymerase (Emerson and Wagner, 1972). This discovery was followed by a set of experiments that enabled the first rescue (reverse genetics system) of VSV defective interfering (DI) particles from cells co-transfected with the five canonical proteins (Pattnaik and Wertz, 1990, 1991). Research on rabies virus (RABV) demonstrated the expression of a reporter gene cloned between the viral 3′ and 5′ termini in cells co-transfected with plasmids encoding for the three helper proteins N, P, and L (Conzelmann and Schnell, 1994). This finding was followed by the recovery of infectious, enveloped rabies virus particles from cDNA (Schnell et al., 1994). Similarly, this strategy was subsequently used to rescue other vertebrate rhabdoviruses, such as VSV (Whelan et al., 1995), Snakehead rhabdovirus (SHRV) (Johnson et al., 2000), infectious haematopoietic necrosis virus (IIHNV) (Brémont, 2005), and haemorrhagic septicaemia virus (VHSV) (Kim et al., 2011).

Similar attempts to reconstitute a plant rhabdovirus started at Jackson’s laboratory, where *N. benthamiana* cellulose-digested protoplasts were found to be suitable for studying sonchus yellow net virus (SYNV) replication (Jones and Jackson, 1990). However, the real challenge was the co-delivery of all plasmids needed for the recovery of recombinant viruses into a single cell. This difficulty was circumvented by using *Agrobacterium* infiltration to co-deliver vectors expressing SYNV helper proteins into *N. benthamiana* leaves (Goodin et al., 2002). In 2013, the same group demonstrated the success of the first plant rhabdovirus SYNV minireplicon (MR) strategy (Ganesan et al., 2013). Plasmids harboring a positive-sense MR cassette containing two sequentially cloned reporter genes between the SYNV 3′ and 5′ termini together with SYNV helper proteins were co-infiltrated into *N. benthamiana*. The fluorescence signal was detected in 5–6 days post-infiltration and was restricted to single cells only. This promising success culminated in 2015 when infectious SYNV was recovered from the upper leaves of *N. benthamiana* plants agroinfiltrated with four plasmids harboring positive-sense SYNV cDNA together with helper protein plasmids (Wang et al., 2015).

### Animal Rhabdoviruses as a Potential Model for Plant Rhabdoviruses in Plant Molecular Pharming

**FIGURE 6:**
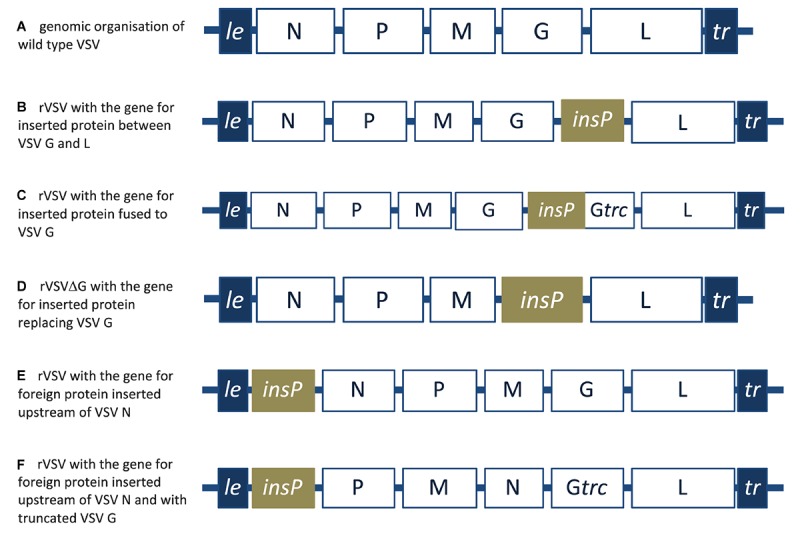
A schematic representation of wild type VSV and recombinant VSV designs. **(A)** 3′ to 5′ negative-strand genomic RNA organization of wild type VSV. **(B)** Recombinant VSV (rVSV) with foreign protein inserted between the VSV G and L genes. **(C)** rVSV with foreign protein fused to the inherent VSV glycoprotein. **(D)** Recombinant VSVΔG with foreign protein replacing the inherent VSV glycoprotein. **(E)** rVSV with foreign protein gene inserted upstream of the VSV N protein. **(F)** rVSV with the gene for foreign protein inserted upstream of the VSV N protein and with truncated VSV glycoprotein. *le*, leader; *tr*, trailer; insP, gene for inserted protein; Gtrc, truncated glycoprotein.

As mentioned earlier, mammalian rhabdoviruses, and in particular vesicular stomatitis virus (VSV), remains the prototype for reverse engineering and molecular adaptation of rhabdoviruses. Like all other rhabdoviruses it has the canonical N, P, M, G, and L genes in a negative sense single stranded RNA genome (Figure 6A). VSV was successfully rescued from cDNA in 1995 (Whelan et al., 1995) and in 1996 it was first engineered to express a reporter gene placed between those of the viral glycoprotein and the polymerase (Schnell et al., 1996b). Later in the same year, recombinant VSV (rVSV) was successfully produced incorporating a foreign protein, in addition to the VSV inherent glycoprotein, in viral envelope (Schnell et al., 1996a). In these recombinant VSV particles, two designs were considered. The first was with the full foreign protein incorporated into the VSV envelope (Figure 6B) while the second was the exoplasmic domain of the foreign protein fused upstream of the transmembrane domain and the cytoplasmic tail of the VSV glycoprotein (Figure 6C). Furthermore, in 1997, recombinant VSV lacking its inherent glycoprotein and expressing CD4 receptor to infect and kill HIV-1 infected T-cells was successfully rescued (Schnell et al., 1997) (Figure 6D).

The ability to rescue VSV without its inherent G protein (VSVΔG) gave the opportunity to generate safer non-propagating VSV based vaccines (Roberts et al., 1999). Moreover, further attenuation strategies based on malleability of the rhabdovirus genome, strategies such as decreasing the expression level of viral genes by manipulating their position down the expression gradient (Flanagan et al., 2003) or by G gene truncations and N gene translocations (Clarke et al., 2007) were also considered. For instance, inserting the foreign gene upstream of the VSV N gene (Figure 6E), or inserting the foreign gene upstream the N gene while relocating the N gene downstream the M gene and truncating VSV G protein (Figure 6F) were also adapted to rescue a recombinant VSV based HIV-1 vaccine (Cooper et al., 2008).

The inherent genomic characteristics of rhabdoviruses, such as having well-defined transcription start/stop signals (Stillman and Whitt, 1997), and the ability to accommodate large inserts while retaining high level expression rates (Haglund et al., 2000), together with negligible seropositivity in the human population (Roberts et al., 1999) made VSV a lucrative candidate for vaccine development and as vector for a number of biomedical applications such as recombinant viral vaccines (Safronetz et al., 2015; Lauretti et al., 2016), gene delivery vectors (Beier et al., 2013), or oncolytic vectors (Bridle et al., 2009) as exemplified in Table 5. Currently, there are a number of on-going clinical trials based on VSV^1^, such as Phase I/II clinical trials for VSV based Ebola virus vaccines (rVSV-ZEBOV) (Huttner et al., 2018).

**TABLE 5:** List of some of the animal rhabdoviruses that have been reverse engineered for various medical applications.

**Recombinant virus vaccine based on VSV**		

	**Pseudotype glycoprotein**	**References**
**Viral vaccines for infectious diseases**		
Ebola virus (EBOV)	Ebola virus Zaire strain (ZEBOV) glycoprotein	Regules et al.,2017
Hepatitis B virus (HBV)	HBV middle envelope surface protein (MS)	Cobleigh et al.,2010
Hepatitis C virus (HCV)	HCV envelope glycoproteins E1 and E2	Majid et al.,2006
Human immunodeficiency virus (HIV)	HIV envelope protein with its cytoplasmic domain replaced with that of the VSV glycoprotein	Rose et al.,2001
Influenza virus	H5N1 (H5 of an H5N1 highly pathogenic avian influenza virus and the N1 of the mouse-adapted H1N1 influenza virus)	Ryder et al.,2015
Severe acute respiratory syndrome (SARS)	SARC coronavirus (CoV) spike (S) glycoprotein	Kapadia et al.,2008
**Oncolytic virotherapy**		
Brain tumor cells	Chikungunya polyprotein (E3-E2-6K-E1)	Zhang et al.,2018
Human T-Cell Leukemia Virus Type 1 (HTLV-1) Infected Cells	HTLV-1 Primary Receptors	Tezuka et al.,2018
Malignant Melanoma	Lymphocytic choriomeningitis virus glycoprotein	Kimpel et al.,2018
Prostate cancer	Lymphocytic choriomeningitis virus glycoprotein	Urbiola et al.,2018
**Biomedical applications**		
Retrograde *trans*-neuronal tracing	Rabies virus glycoprotein	Beier et al.,2013

Nowadays VSV is commonly considered a very successful and widely used platform for heterologous expression of more complex and interesting pharmaceuticals and biomolecules, and for the generation of (pseudo) typed eVLPs for vaccine purposes. It is expected that the availability and exploitation of enveloped plant viruses as biological particles, in analogy to VSV, will give a boost to the exploitation of plant-based production platforms for more complex (glyco)proteins.

## Concluding Remarks

Plant molecular pharming has materialized as a reliable and cost-effective platform for the production of pharmaceuticals, vaccines, and biobetter products with a number of plant-produced proteins starting to be commercialized (Ratner, 2010; Yusibov et al., 2011). Simultaneously, the development of viral vectors, together with agroinfection, culminated in rapid transient expression systems that successfully express high levels of large and complex pharmaceutical proteins and antimicrobial peptides (Fischer and Emans, 2000; Chen et al., 2013; Peyret and Lomonossoff, 2013; Loh et al., 2017; Leite et al., 2019) and enabled the production of plant-virus based VLPs against chronic and infectious diseases (Hefferon, 2018; Sahithi et al., 2019). However, as with their comparative systems counterparts, plant-virus based VLP(s) are inherently constrained in their ability to present complex antigens and glycoproteins. Moreover, in the case of rapidly evolving viruses, such as influenza, selective pressure will drive viral evasion of immune response induced by VLP vaccines based on few amino acids or antigens that cannot reproduce native conformational epitopes. Furthermore, attempts to produce plant-made eVLP, such as influenza HA VLP, has been so far proven successful only because of the inherent characteristics of the influenza virus HA and its ease of detachment as eVLP from plants cell surface (D’Aoust et al., 2010); a basis for success not necessary applicable in other cases. Therefore, developments in reverse engineering inherently enveloped plant-viruses such as rhabdoviruses is expected to give plant molecular pharming a platform for a wide range of biotechnological applications, most relevant of which is expressing enveloped VLPs exposing chimeric and complex antigens.

Considering VSV as prototype, the inherent rhabdovirus characteristics of being enveloped, with defined genomic transcription units (Schnell et al., 1996b), genome stability (Walker et al., 2015), and an ability to stably incorporate recombinant glycoprotein into their envelope (Schnell et al., 1996a) make them ideal for various biotechnological applications. Plant rhabdoviruses, share such inherent characteristics, together with dispensability of the G protein for systemic plant-infection as demonstrated with SNYV (Wang et al., 2015). The G protein is required for virus entry and subsequent propagation within the arthropods vector (Hogenhout et al., 2003), while only the carboxyl-terminal domain of the G protein, together with M protein are required for the morphogenesis and the budding of the enveloped particles (Sun et al., 2018). Hence these additional characteristics will enable large scale production of recombinant rhabdovirus based eVLP in which the (exposed) ectodomain of the G has been exchanged for another gene-of-interest (GOI) glycoprotein. Such chimeric viruses are contained within the plant and require less stringent containment controls. Similarly to VSV-recombinant design, plant rhabdovirus vector constructs can also be constructed containing a GOI at different gene positions for various expression purposes (Figure 6). All these advantages together with the establishment of plant molecular pharming as cost effective and reliable production platform, make plant rhabdoviruses promising candidates in biotechnology in general and in the field of plant-made recombinant viral vaccines in specific.

## Author Contributions

AI wrote the first draft of the manuscript. AI, VO, and RK contributed to manuscript revision, and also read and approved the submitted version.

## Conflict of Interest Statement

The authors declare that the research was conducted in the absence of any commercial or financial relationships that could be construed as a potential conflict of interest.

## Footnotes

1 https://clinicaltrials.gov
